# MiR-132-3p Modulates MEKK3-Dependent NF-κB and p38/JNK Signaling Pathways to Alleviate Spinal Cord Ischemia-Reperfusion Injury by Hindering M1 Polarization of Macrophages

**DOI:** 10.3389/fcell.2021.570451

**Published:** 2021-02-11

**Authors:** Hua Fang, Hua-Feng Li, Qin Pan, Hon-Ling Jin, Miao Yang, Ru-Rong Wang, Quan-Yun Wang, Jian-Ping Zhang

**Affiliations:** ^1^Department of Anesthesiology, Guizhou Provincial People’s Hospital, Guiyang, China; ^2^Department of Anesthesiology, Guizhou University People’s Hospital, Guiyang, China; ^3^Laboratory of Anesthesiology and Perioperative Medicine, Guizhou University School of Medicine, Guiyang, China; ^4^Department of Anesthesiology, West China Second University Hospital, Sichuan University, Chengdu, China; ^5^Department of Anesthesiology, West China Hospital, Sichuan University, Chengdu, China

**Keywords:** spinal cord ischemia-reperfusion injury, microRNA-132-3p, MEKK3, NF-κB, p38/JNK, macrophage

## Abstract

Spinal cord ischemia-reperfusion (SCIR) injury is a serious complication of open surgical and endovascular aortic procedures. MicroRNA-132-3p (miR-132-3p) has been reported to be involved in the progression of various diseases, but its role in SCIR injury is unclear. Thus, we aimed in this study to investigate the mechanism of miR-132-3p in SCIR injury and explore its pathway as a therapeutic target for SCIR injury. We first constructed a SCIR injury rat model and documented motor function in the model. Reverse transcription quantitative polymerase chain reaction (RT-qPC)R and Western blot analysis were used to detect the expression of miR-132-3p and mitogen-activated protein kinase kinase kinase 3 (MEKK3) in SCIR injury rats. The interaction between miR-132-3p and MEKK3 was identified by dual-luciferase reporter gene assay. Then, the effects of miR-132-3p and MEKK3 on macrophage M1 polarization were evaluated *in vitro* and *in vivo* by altering their expression in macrophages of SCIR injury rats, with treatments altering the nuclear factor-kappaB (NF-κB) and c-Jun N-terminal kinase (JNK)/p38 signaling pathways using SP600125, SB203580, or PDTC. The SCIR injury rats had a high Tarlov score and low miR-132-3p expression along with high MEKK3 expression. miR-132-3p could directly bind to MEKK3, and that macrophage M1 polarization and inflammation could be inhibited by overexpression of miR-132-3p through downregulating MEKK3 and inactivating the NF-κB and p38/JNK signaling pathways. Besides, increased miR-132-3p expression could decrease the injured rat Tarlov score. Overall, our study demonstrated that miR-132-3p can suppress M1 polarization of macrophages and alleviate SCIR injury by blocking the MEKK3-dependent activation of the NF-κB and p38/JNK signaling pathway. Thus, miR-132-3p and its downstream pathways may be useful targets to alleviate the symptoms of SCIR injury.

## Introduction

Spinal cord ischemia-reperfusion (SCIR) injury, may result from spinal cord blood flow interruption during aortic surgery, which occurs post-operatively with an incidence rate of 28% (Zhu et al., 2013). Acute SCIR injury is related to some pathologic changes such as inflammation, neuronal apoptosis, and edema (Ning et al., 2012), and can give rise to neurological deficits such as acute or delayed paraplegia (Wyndaele, 2016). Macrophages have been identified to participate in the development of various inflammatory diseases including cancer, fibrosis, and cardiovascular disease (Meli et al., 2019). The inflammation caused by activation of macrophages and resident microglia plays a key role in spinal cord injury progression (David and Kroner, 2011). Furthermore, it has been documented that macrophages primarily remain in the proinflammatory M1 state in the injured spinal cord (Kroner et al., 2014). Various approaches have been exploited to improve spinal ischemia tolerance, such as bypass grafting, and hypothermia (Fang et al., 2013). However, effective procedures for the prevention and treatment of SCIR injury have not been well defined (Yin et al., 2014), which motivates our search for new therapeutic avenues.

MicroRNAs (miRNAs or miRs) are small non-coding RNA molecules that can directly bind to target mRNAs and modulate their expression at the post-transcriptional level, and which participate in the development of diverse diseases, including SCIR injury (Li et al., 2015). Besides, ischemia-reperfusion plays a role in regulating the expression of miRNAs and leads to severe inflammation (Li et al., 2017). As such, miR-22-3p may play sustained regulatory roles in SCIR injury through inflammatory pathways (Liu et al., 2020). Furthermore, miR-496 served as a suppressor in SCIR injury through its inhibitory effects on inflammation and apoptosis (Xu et al., 2016). Previous work by Leinders et al. (2016) showed that the expression of miR-132-3p could be upregulated in spinal and dorsal root ganglia by spared nerve injury. Moreover, we predicted that miR-132-3p could bind to mitogen-activated protein kinase kinase kinase 3 (MEKK3), which plays a role as an activator of some signaling pathways through formation of particular signaling modules (Cullere et al., 2015). Another study showed that MEKK3 silencing had protective effects against spinal cord injury (Kong et al., 2019), whereas other work showed that MEKK3 could be targeted specifically by protein phosphatase 2A (PP2A) to suppress the activation of the nuclear factor-kappaB (NF-κB) signaling pathway (Sun et al., 2010). Furthermore, reduced expression of MEKK3 in microglia could lead to inactivation of the NF-κB signaling pathway, which is an essential transcription factor that modulates inflammatory responses by influencing the expression of pro-inflammatory factors including tumor necrosis factor-α (TNF-α), inducible nitric oxide synthase (iNOS), and interleukin-6 (IL-6) (Yao et al., 2018). Another study indicated that enhancement of the c-Jun N-terminal kinase (JNK)/p38 signaling pathway contributes to a remarkable increase of neuron apoptosis and stronger motor deficits following ischemia-reperfusion *in vivo* as well as neuron injury *in vitro* (Chen et al., 2018). Based on a compilation of these previous accounts, we hypothesized that miR-132-3p may participate in the development of SCIR injury by interacting with MEKK3 and the NF-κB and p38/JNK signaling pathways. The present study was performed to test our hypothesis using *in vitro* and *in vivo* assays.

## Materials and Methods

### Ethics Statement

The study protocol was approved by the Experimental Animal Ethics Committee of Guizhou Provincial People’s Hospital. Animal experiment strictly followed the principle to minimize the pain, suffering, and discomfort to experimental animals.

### Construction and Identification of the SCIR Injury Rat Model

Forty-five Sprague Dawley rats aged 8 weeks and weighing 200–250 g were provided by Guizhou Laboratory Animal Engineering Technology Center (Guizhou, China). Rats were acclimated in a standard cage for 1 week before surgery with free access to food and water. The rats were raised in a light/dark cycle of 12/12-h at 22–24°C, with a relative humidity of 50–60%. Then 37 of the rats were randomly chosen to construct the model of SCIR injury. Here, the rats were anesthetized with intraperitoneal injection of 4% pentobarbital sodium (50 mg/kg), and then endotracheally intubated and mechanically ventilated. The arcus aortae were exposed by a cervicothoracic incision, and ischemia was induced by placing a vascular clamp between the left carotid artery and the left subclavian artery. Effective ischemia was maintained for 12 min and a Doppler monitor (Moor Instruments, Axminster, United Kingdom) was used to confirm that blood flow in the caudal artery decreased by 90%. Then the clamp was removed and the rats were recovered with reperfusion for 48 h. The sham-operated rats were subjected to the same procedure without clamping. A total of 28 rats were successfully modeled, among which eight served as the SCIR injury group.

The synthetic miR-132-3p agomir and negative control (NC) agomir plasmids (purchased from Dharmacon, Inc., Chicago, IL, United States) were administered intrathecally into the rats using a 20 μL microsyringe (Gaoge Co., Ltd., Shanghai, China) at the L_5__–__6_ spinal segment three times (15 μL each time; 100 μM) at intervals of 24 h (Li et al., 2016; Zhang et al., 2018), with nine rats for each group.

### Motor Behavioral Assessment

The hind-limb motor functions were assessed by Tarlov scores ranging from 0 (no ankle movement) to 4 (normal). The assessment was done on days 1, 3, 7, and 14 after surgery by two independent observers who were blinded to the experimental conditions (Zhang et al., 2018).

### Hematoxylin-Eosin Staining

Rats were intraperitoneally anesthetized with 4% pentobarbital sodium (50 mg/kg) and perfused transcardially with 0.9% NaCl, followed by 4% paraformaldehyde in 0.01 M phosphate buffered saline (PBS, pH = 7.4). Spinal cord tissues were cut at 0.5 cm on each side of the injury and embedded in paraffin. The slices were warmed for 1 h at 60°C, conventionally deparaffinized, hydrated with gradient alcohol, and stained with hematoxylin solution (Beijing Solarbio Science and Technology Co., Ltd., Beijing, China) for 2 min, and rinsed with tap water for 10 s, followed by color separation with 1% hydrochloric acid-alcohol for 10 s. Then, slices were stained by eosin staining solution for 1 min and then washed with distilled water for 1 min. The slices were then dehydrated with gradient alcohol, cleared with xylene, and sealed with neutral balsa for examination of morphological characteristics under an optical microscope (XP-330, Shanghai Bingyu optical instrument Co., Ltd., Shanghai, China) (Xu et al., 2018).

### Immunohistochemistry

Paraffin-embedded tissue slices were rehydrated with gradient alcohol, and soaked in 3% H_2_O_2_ in methanol for 20 min, followed by antigen retrieval in a water bath. The tissue slices were incubated with normal goat serum blocking solution (C-0005, Shanghai Haoran Biologic Technology Co., Ltd., Shanghai, China) at room temperature for 20 min and then the excess liquid on the slice was removed. Afterward, the tissue slices were incubated with primary antibody rabbit anti-CD68 (ab125212, 1:100, Abcam, Cambridge, United Kingdom) overnight at 4°C, washed with 0.1 M PBS, and further incubated with secondary antibody of goat anti-rat immunoglobulin G (IgG) (ab6785, 1:1,000, Abcam, Cambridge, United Kingdom) at 37°C for 20 min. Then slices were washed with 0.1 M PBS and incubated with streptomycin albumin working solution labeled by horseradish peroxidase (HRP) at 37°C for 20 min. Then, the slices were washed with 0.1 M PBS and colored with diaminobenzidine, followed by counterstaining with hematoxylin (PT001, Shanghai Bogoo Biotechnology. Co., Ltd., Shanghai, China) for 1 min. Then slices were blued in 1% ammonia water, washed by water, dehydrated by gradient alcohol, cleared with xylene, mounted with neutral resin, and observed under a microscope.

### Macrophage Collection, Culture, and Transfection

The isolated spinal L_4__–__6_ segments were rinsed with sterile calcium- and magnesium-free Hanks’ balanced salt solution. The tissues were harvested with 3 mL Roswell Park Memorial Institute (RPMI) 1,640 medium containing 5 mg/mL bovine serum albumin (BSA), 100 U/mL penicillin, 100 μg/mL streptomycin and 1% glutamine. Then the suspension was transferred to 15 mL conical propylene-coated tubes (Falcon Blue Max Jr) and centrifuged for 30 s to separate the supernatant (containing macrophages) from the tissues. After that, supernatant was transferred to a fresh tube, added with 5 mL medium and kept on ice. Next, the mixture was divided into two equal parts (about 2.5 mL each), transferred into two 35-mm polystyrene cellular wells (Corning, NY, United States), and cultured with 5% CO_2_ for 2 h at 37°C. The supernatant was discarded and adherent macrophages were cultured in 3 mL freshly prepared culture medium containing 10 μg/mL of lipopolysaccharide (LPS) (055: B5 *Escherichia coli*, Sigma-Aldrich, Inc., St. Louis, MO, United States). Following the instructions of Lipofectamine 2,000 reagents (Invitrogen, Thermo Fisher Scientific, CA, United States), cells were separated into different groups based on the plasmids used for cell transfection, namely with NC mimic, miR-132-3p mimic, NC inhibitor, miR-132-3p inhibitor, si-NC, and si-MEKK3. In addition, cells were also added with 10 μM dimethyl sulfoxide (DMSO), 10 μM SP600125 (an inhibitor of the JNK signaling pathway; HY-12041, MedChem Express, NJ, United States), 10 μM SB203580 (an inhibitor of the p38 signaling pathway; HY-10256, MedChem Express, NJ, United States), or 10 μM pyrrolidine dithiocarbamate (PDTC) (an inhibitor of the NF-κB signaling pathway; HY-18738, MedChem Express, NJ, United States).

### Immunofluorescence Staining

The frozen slices or cells on cover glass were permeabilized by 0.1 M Tris–HCl buffer (pH = 7.6) containing 0.3% Triton. The slices or cells were incubated with fluorescent primary antibody for MEKK3 (ab40756, 1:100, Abcam, Cambridge, United Kingdom), CD68 (ab125212, 1:100, Abcam, Cambridge, United Kingdom), iNOS (ab178945, 1:250, Abcam, Cambridge, United Kingdom), and arginase-1 (Arg-1) (ab91279, 1:250, Abcam, Cambridge, United Kingdom) overnight at 4°C. Then, slices or cells were washed with PBS, incubated with fluorescence secondary antibody (1:500) in the dark for 2 h at room temperature and incubated with 4′,6-diamidino-2-phenylindole (ab104139, 1:100, Abcam, Cambridge, United Kingdom) in the dark for 10 min at room temperature. Finally, slices or cells were washed by PBS, mounted with mounting medium and observed under a fluorescence microscope.

### Reverse Transcription Quantitative Polymerase Chain Reaction

Total RNA of cells or tissues was extracted using TRIzol reagents (15596026, Invitrogen, Thermo Fisher Scientific, CA, United States) and reversely transcribed into complementary DNA (cDNA) according to the instructions of the EasyScript First-Strand cDNA Synthesis SuperMix (AE301-02, TransGen Biotech Co., Ltd., Beijing, China) and Mir-X^TM^ miRNA qRT-PCR SYBR^®^ kits (638314, Takara Biomedical Technology (Beijing) Co., Ltd., Beijing, China). These synthetic cDNAs were then subjected to reverse transcription quantitative polymerase chain reaction (RT-qPCR) using Fast SYBR Green PCR kit (Applied Biosystems, Thermo Fisher Scientific, CA, United States) and the ABI 7500 RT-PCR system (Applied Biosystems, Thermo Fisher Scientific, CA, United States). Glyceraldehyde-3-phosphate dehydrogenase (GADPH) was used as an internal reference. The relative expression of target genes was calculated by the 2^–ΔΔ*Ct*^ method. The primer sequences are shown in Table 1.

**TABLE 1:** Primer sequences for RT-qPCR.

Target	Primer sequences (5′–3′)
miR-132-3p	F: 5′-TGCGCTAACAGTCTACAGCCAT-3′
	R: 5′-CCAGTGCAGGGTCCGAGGTATT-3′
MEKK3	F: 5′-TGGATGAACAAGAGGCATTAGACT-3′
	R: 5′-CATATCCAGACACCCGGGGA-3′
iNOS	F: 5′-CTTTTAGAGACGCTTCTGAG-3′
	R: 5′-TTTGATGCTTGTGACTCTTA-3′
TNF-α	F: 5′-CCTCTTCTCATTCCTGCTC-3′
	R: 5′-CTTCTCCTCCTTG TTGGG-3′
Arg-1	F: 5′-CCGCAGCATTAAGGAAAGC-3′
	R: 5′-CCCGTGGTCTCTCACATTG-3′
IL-10	F: 5′-GCACTGCTATGTTGCCTGCT-3′
	R: 5′-TCAGCTCTCGGAGCATGTG-3′
CD86	F: 5′- ACCAGGCTCTACGACTTCAC-3′
	R: 5′- GACCAGCAGAAAGAGACAGC-3′
CD206	F: 5′-GAGGACTGCGTGGTGATGAA-3′
	R: 5′- CAGCGAACGTTGAAAGGGTG-3′
U6	F: 5′-CTCGCTTCGGCAGCACA-3′
	R: 5′-AACGCTTCACGAATTTGCGT-3′
GAPDH	F: 5′-ACTGCCACTCAGAAGACTGT-3′
	R: 5′-TGCTGTAGCCATATTCATTG-3′

*RT-qPCR, reverse transcription quantitative polymerase chain reaction; F, forward; R, reverse; miR-132-3p, microRNA-132-3p; MEKK3, mitogen-activated protein kinase kinase kinase3; iNOS, inducible nitric oxide synthase; TNF-α, tumor necrosis factor-α; Arg-1, arginase-1; IL-10, interleukin-10; GAPDH, glyceraldehyde-3-phosphate dehydrogenase.*

### Western Blot Analysis

Total protein of the cell was extracted by radio-immunoprecipitation assay (RIPA) lysis buffer (BB-3209, Bestbio, Shanghai, China). After separation by sodium dodecyl sulfate-polyacrylamide gel electrophoresis, proteins were transferred onto polyvinylidene fluoride membranes. After being blocked for 1 h, the membrane was incubated at 37°C for 1 h with the following primary rabbit polyclonal antibodies: MEKK3 (ab40756, 1:1,000), JNK (ab179461, 1:1,000), phosphorylated JNK (ab124956, 1:1,000), p38 (ab170099, 1:5,000), phosphorylated p38 (ab4822, 1:1,000), IκB kinase complex (IKK-β) (ab124957, 1:1,000), and phosphorylated IKK-β (ab59195, 1:500). Then, the membrane was incubated with HRP-conjugated secondary goat anti-rabbit IgG (ab205718, 1:20,000). The above antibodies were from Abcam (Cambridge, United Kingdom). Subsequently, the membrane was washed with PBS and visualized. The ratio of the gray value of the target band to GAPDH was representative of the relative protein expression.

### Enzyme-Linked Immunosorbent Assay

The supernatant was collected from the cultured cells in each group. According to the specifications of the manufacturer, the concentrations of interleukin-10 (IL-10) and TNF-α were measured by enzyme-linked immunosorbent assay (ELISA) kit (R&D Systems, Minneapolis, MN, United States). The optical density (OD) value was measured at 450 nm. Standard curves made from standard samples of IL-10 and TNF-α were used to calculate cytokine production.

### Flow Cytometry

Cells were washed with PBS containing 5% BSA and then incubated with antibodies for fluorescent dye coupling: anti-CD86 and anti-CD206 (eBioSCIRence Inc., San Diego, CA, United States). Dead cells were identified by propyl iodide staining. The samples were evaluated by a FACSCanto II device using FACS Diva software (BD Biosciences, Heidelberg, Germany) (Li et al., 2016).

### Dual-Luciferase Reporter Gene Assay

The target gene of miR-132-3p was predicted using a biological prediction website. The sequence of MEKK3 3′untranslated region (3′UTR) was amplified by PCR and the target segment was cloned to the downstream of luciferase reporter gene pmirGLO (3577193, Promega, Madison, WI, United States) using *Xho*I and *Not*I cleavage sites, with pMEKK3-wild type (WT) as the vector. Then, site-directed mutagenesis was conducted for the MEKK3 binding site and pMEKK3-mutant type (MUT) vector was constructed. Thereafter, the two plasmids were co-transfected with miR-132-3p mimic and NC mimic plasmids separately into human embryonic kidney 293T (HEK-293T) cells. After 24 h, cells were lysed and centrifuged at 12,000 rpm for 1 min to collect the supernatant. Luciferase activity was measured by Dual-Luciferase^®^ Reporter Assay System (E1910, Promega Corporation, Madison, WI, United States). Firefly luciferase was detected by adding 100 μL firefly luciferase working fluid to cell samples and 100 μL renilla luciferase working fluid was added to the cell samples to detect the renilla luciferase activity. The ratio of firefly luciferase activity to renilla luciferase activity indicated the relative luciferase activity. In order to observe the activity of NF-κB, 0.5 μg NF-κB reactive luciferase reporter plasmid containing 4 κB sites (pNF-κB-Luc; Clontech, Takara Bio Inc., Tokyo, Japan) and 0.2 μg pSV-β-galactosidase expression plasmid (Promega, Madison, WI, United States) were co-transfected to cells in a 12-well plate. After 24 h, cells were subjected to different treatments and luciferase activity was analyzed by luminometer and standardized using the β-galactosidase activity.

### Statistical Analysis

Statistical analysis was conducted using SPSS 21.0 statistical software (IBM Corp. Armonk, NY, United States). Measurement data were expressed as mean ± standard deviation. Differences of unpaired-designed data between two groups were compared by unpaired *t*-test. Comparisons of data among multiple groups were conducted by one-way analysis of variance (ANOVA) with *post hoc* comparisons by Student Newman–Kuels tests. The data of Tarlov score were analyzed by repeated measures ANOVA followed by Bonferroni *post hoc* tests with corrections for intra-group comparisons. A value of *p* < 0.05 indicated that the difference was statistically significant.

## Results

### Expression of MiR-132-3p Is Downregulated in Spinal Cord Tissues of SCIR Injury Rats

**FIGURE 1:**
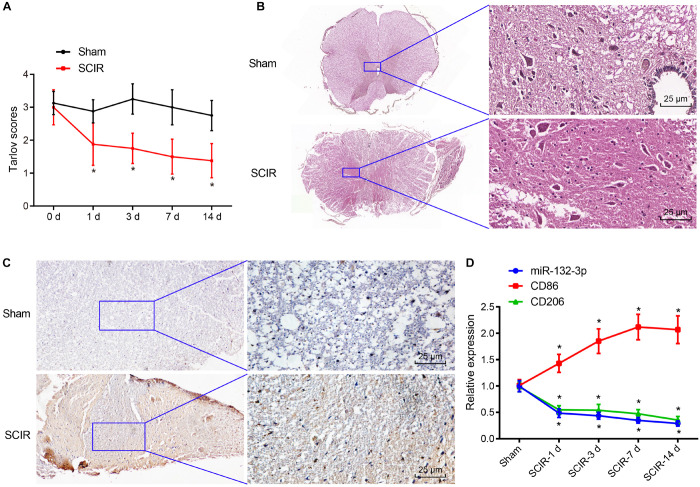
Spinal cord tissues of SCIR injury rats exhibit low expression of miR-132-3p. **(A)** Tarlov score of SCIR injury rats and sham-operated rats. **(B)** Pathological features of spinal cord neurons in spinal cord tissues of SCIR injury rats and sham-operated rats observed by HE staining. **(C)** Expression of CD68 (a macrophage marker) in spinal cord tissues of SCIR injury rats and sham-operated rats detected by immunohistochemistry. **(D)** Expression of miR-132-3p, CD206, and CD86 in spinal cord tissues of SCIR injury rats and sham-operated rats measured by RT-qPCR. *p < 0.05 vs. sham-operated rats. Measurement data were expressed as mean ± standard deviation. *n* = 8 for rats following each treatment. Comparisons of data in panel **(A)** were conducted by repeated measures ANOVA and those in panel **(D)** were conducted by one-way ANOVA.

After construction of the SCIR injury rat model, the motor behavior of SCIR injury rats and sham-operated rats was evaluated using Tarlov assessment. The results showed that the average score of SCIR injury rats was lower than that of sham-operated rats, and the average score was the lowest at the 14th day (*p* < 0.05) (Figure 1A). This suggested that motor behavior of rats became gradually worse with increasing time after ischemia reperfusion and that the rat model of SCIR injury was constructed successfully. Analysis using hematoxylin-eosin (HE) staining showed that the spinal cord neurons of spinal cord tissues of sham-operated rats were clear and normal, while cellular swelling and bleeding caused by widen surrounding gap, and vacuole formation were found in the spinal cord neurons of SCIR injury rats (Figure 1B). Moreover, immunohistochemistry indicated an increase of the expression of macrophage marker CD68 in spinal cord tissues of SCIR injury rats in comparison with sham-operated rats (*p* < 0.05) (Figure 1C), which suggested the presence of macrophage infiltration at the site with SCI. At the same time, the expression of miR-132-3p and CD206 (a typical M2 macrophage marker) was decreased with increasing time after in the injury, while that of CD86 (a typical M1 macrophage marker) was increased significantly at the 1st day after injury and then decreased gradually (Figure 1D). These results indicated a potential correlation between miR-132-3p expression and motor impairment of rats in the SCIR model. The expression of miR-132-3p and CD206 was similar, which showed that miR-132-3p may be specifically expressed in M2 macrophages during SCIR injury.

### Elevated MiR-132-3p Suppresses M1 Polarization of Macrophages Isolated From SCIR Injury Rats

**FIGURE 2:**
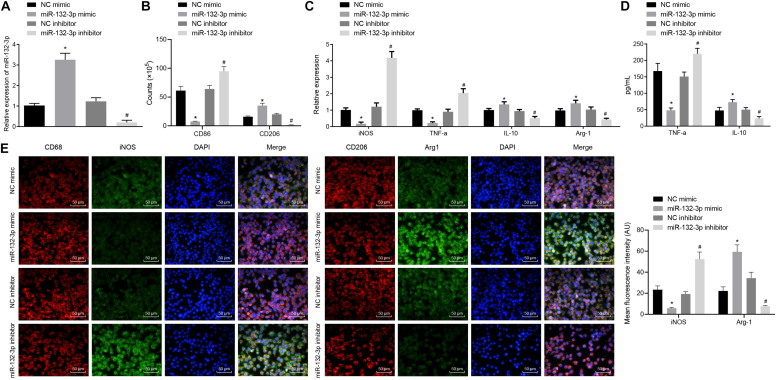
miR-132-3p inhibits M1 polarization of macrophages from SCIR injury rats. The macrophages from SCIR injury rats were transfected with miR-132-3p mimic, miR-132-3p inhibitor, or their corresponding NC plasmids. **(A)** Expression of miR-132-3p in macrophages detected by RT-qPCR. **(B)** Expression of CD86 (a specific markers of M1 phenotype) and CD206 (a specific marker of M2 phenotype) in macrophages detected by flow cytometry. **(C)** Expression of iNOS, TNF-α, IL-10, and Arg-1 in macrophages measured by RT-qPCR. **(D)** Contents of TNF-α and IL-10 in macrophages determined by ELISA. **(E)** Expression of iNOS and Arg-1 in macrophages measured by immunofluorescence staining. *p < 0.05 vs. macrophages transfected with NC mimic. #*p* < 0.05 vs. macrophages transfected with NC inhibitor. Measurement data were expressed as mean ± standard deviation. Differences between two groups were compared by unpaired *t*-test and those among multiple groups were compared by one-way ANOVA. The experiment was repeated three times independently.

To observe the effects of miR-132-3p on macrophage polarization in SCIR injury, the macrophages from SCIR injury rats were separately transfected with miR-132-3p mimic, miR-132-3p inhibitor, or their respective NC plasmids. The results from RT-qPCR revealed that the expression of miR-132-3p was higher in macrophages transfected with miR-132-3p mimic while miR-132-3p inhibitor inhibited its expression (Figure 2A). Flow cytometric analysis displayed that, in macrophages transfected with miR-132-3p mimic, the proportion of cells expressing CD86 (a specific marker of M1 phenotype) was decreased, while the proportion of cells expressing CD206 (a specific marker of M2 phenotype) was increased. Conversely, miR-132-3p inhibitor brought about opposite effects (Figure 2B). In addition, the expression of iNOS (a specific marker of M1 phenotype) and TNF-α was found to be downregulated, while that of IL-10 and Arg-1 (a specific marker of M2 phenotype) was upregulated in macrophages transfected with miR-132-3p mimic. miR-132-3p inhibitor led to an increase of iNOS and TNF-α expression, yet a decline of IL-10 and Arg-1 expression (Figure 2C). The results of ELISA showed that the level of TNF-α was reduced and the level of IL-10 was increased in macrophages transfected with miR-132-3p mimic, which was negated by miR-132-3p inhibitor (Figure 2D). Immunofluorescence staining showed that the treatment of miR-132-3p mimic inhibited the expression of iNOS and promoted the expression of Arg-1, which was opposite to the effects of miR-132-3p inhibitor (Figure 2E). These results demonstrated that overexpression of miR-132-3p could inhibit M1 polarization of macrophages from SCIR injury rats.

### MEKK3 Is Highly Expressed in Spinal Cord Tissues of SCIR Injury Rats and Is a Target Gene of MiR-132-3p

**FIGURE 3:**
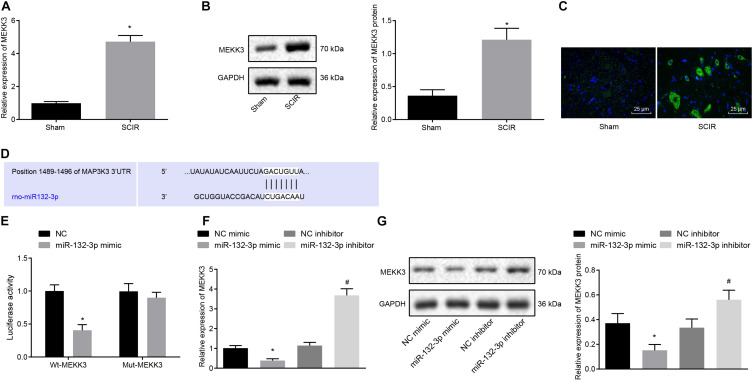
Spinal cord tissues of SCIR injury rats present with high expression of MEKK3 which is a target gene of miR-132-3p. **(A)** mRNA expression of MEKK3 in spinal cord tissues of sham-operated rats and SCIR injury rats measured by RT-qPCR. **(B)** Protein expression of MEKK3 in spinal cord tissues of sham-operated rats and SCIR injury rats measured by Western blot analysis; *n* = 8. **(C)** Expression of MEKK3 and CD68 in spinal cord tissues of sham-operated rats and SCIR injury rats detected by immunofluorescence staining. **(D)** Binding sites of miR-132-3p to the 3′UTR of MEKK3 mRNA predicated by a bioinformatics database. **(E)** Luciferase activity of cells co-transfected with miR-132-3p mimic and MEKK3-WT or MEKK3-MUT detected by dual-luciferase reporter gene assay. **(F)** mRNA expression of MEKK3 in cells treated with miR-132-3p mimic, miR-132-3p inhibitor, or their NC plasmids detected by RT-qPCR. **(G)** Protein expression of MEKK3 in cells treated with miR-132-3p mimic, miR-132-3p inhibitor, or their NC plasmids measured by Western blot analysis. ^∗^*p* < 0.05 vs. macrophages transfected with NC mimic. #*p* < 0.05 vs. macrophages transfected with NC inhibitor. *n* = 8 for rats following each treatment. Measurement data were expressed as mean ± standard deviation. Differences between two groups were compared by unpaired *t*-test and those among multiple groups were compared by one-way ANOVA. The experiment was repeated three times independently.

A recent study has shown that silencing of MEKK3 can reduce cardiomyocyte injury caused by hypoxia/reoxygenation (Wang et al., 2019), and that MEKK3 can induce the secretion of macrophage-related inflammatory factors (Gillis and Keashly, 1991). In the present study, we aimed to validate the expression of MEKK3 in SCIR injury. The results from RT-qPCR and Western blot analysis suggested that the mRNA and protein expression of MEKK3 was higher in spinal cord tissues of SCIR injury rats than in sham-operated rats (*p* < 0.05) (Figures 3A,B). Meanwhile, immunofluorescence staining also confirmed the increased fluorescence activity of MEKK3 in spinal cord tissues of SCIR injury rats (*p* < 0.05) (Figure 3C). A bioinformatics database^1^ predicted a specific binding site between MEKK3 and miR-132-3p (Figure 3D). Dual-luciferase reporter gene assay further verified the binding, in showing that the luciferase activity of MEKK3-WT was decreased (*p* < 0.05), while that of MEKK3-MUT showed no alterations in cells co-transfected with miR-132-3p mimic (*p* > 0.05) (Figure 3E). Furthermore, the results from RT-qPCR and Western blot analysis indicated a downward trend in the MEKK3 expression in macrophages transfected with miR-132-3p mimic, while an upward trend was evident in the MEKK3 expression following miR-132-3p silencing (*p* < 0.05) (Figures 3F,G). Taken together, miR-132-3p could target MEKK3 and negatively regulate its expression.

### MiR-132-3p Negatively Regulates MEKK3 to Suppress M1 Polarization of Macrophages From SCIR Injury Rats

**FIGURE 4:**
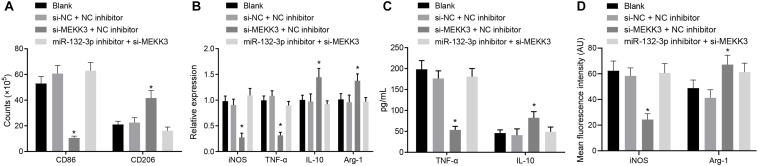
miR-132-3p inhibits the M1 polarization of macrophages from SCIR injury rats by down-regulating MEKK3. The macrophages were transfected with both si-NC and NC inhibitor, both si-MEKK3 and NC inhibitor, or both miR-132-3p inhibitor and si-MEKK3. **(A)** Expression of CD86 (a specific marker of M1 phenotype) and CD206 (a specific marker of M2 phenotype) in macrophages detected by flow cytometry. **(B)** Expression of inflammatory factors iNOS, TNF-α, IL-10, and Arg-1 in macrophages detected by RT-qPCR. **(C)** The level of TNF-α and IL-10 in macrophages measured by ELISA. **(D)** Expression of iNOS and Arg-1 in macrophages detected by immunofluorescence staining. *p < 0.05 vs. macrophages transfected with both si-NC and NC inhibitor. Measurement data were expressed as mean ± standard deviation. Comparisons of data among multiple groups were conducted by one-way ANOVA. The experiment was repeated three times independently.

In order to verify that miR-132-3p regulates macrophage polarization through MEKK3, macrophages were separately transfected with both si-NC and NC inhibitor, both si-MEKK3 and NC inhibitor, or both miR-132-3p inhibitor and si-MEKK3. Flow cytometric data showed that, compared with macrophages transfected with both si-NC and NC inhibitor, the proportion of cells expressing CD86 was decreased, but that of CD206 was increased in macrophages transfected with both si-MEKK3 and NC inhibitor (*p* < 0.05) (Figure 4A). The results of RT-qPCR showed that the expression of iNOS and TNF-α was decreased while that of IL-10 and Arg-1 was increased in macrophages co-transfected with si-MEKK3 and NC inhibitor in comparison with the macrophages co-transfected with si-NC and NC inhibitor (*p* < 0.05) (Figure 4B). In addition, the results of ELISA indicated that the level of TNF-α was reduced whereas that of IL-10 was increased in response to co-treatment of si-MEKK3 and NC inhibitor relative to the co-treatment of si-NC and NC inhibitor (Figure 4C). The results of immunofluorescence staining suggested that iNOS expression was downregulated and Arg-1 expression was upregulated in cells co-transfected with si-MEKK3 and NC inhibitor vs. cells co-transfected with si-NC and NC inhibitor (*p* < 0.05) (Figure 4D). All in all, miR-132-3p elevation could inhibit M1 polarization of macrophages by reducing the expression of MEKK3.

### MiR-132-3p Downregulates MEKK3 to Block Activation of the NF-κB and p38/JNK Signaling Pathways and Further Attenuates M1 Polarization of Macrophages From SCIR Injury Rats

**FIGURE 5:**
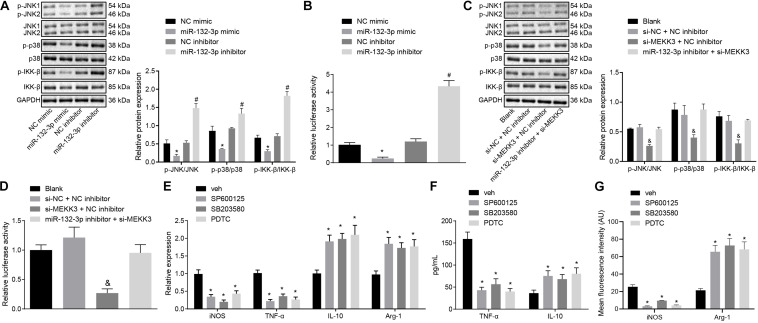
miR-132-3p upregulation hinders M1 polarization of macrophage from SCIR injury rats by inactivating the NF-κB and p38/JNK signaling pathways *via* MEKK3 downregulation. **(A)** Protein expression of JNK, p38, and IKK-β as well as the extent of JNK, p38, and IKK-β phosphorylation measured by Western blot analysis in macrophages transfected with miR-132-3p mimic or miR-132-3p inhibitor. **(B)** NF-κB luciferase activity detected by dual-luciferase reporter gene assay in macrophages transfected with miR-132-3p mimic or miR-132-3p inhibitor. **(C)** Protein expression of JNK, p38, and IKK-β as well as the extent of JNK, p38, and IKK-β phosphorylation measured by western blot analysis in macrophages transfected with si-MEKK3 or in combination with miR-132-3p inhibitor. **(D)** NF-κB luciferase activity detected by dual-luciferase reporter gene assay in macrophages transfected with si-MEKK3 or in combination with miR-132-3p inhibitor. *p < 0.05 vs. macrophages transfected with NC mimic. #*p* < 0.05 vs. macrophages transfected with NC inhibitor. &*p* < 0.05 vs. macrophages transfected with both si-NC and NC inhibitor. **(E)** Expression of inflammatory factors iNOS, TNF-α, IL-10, and Arg-1 in macrophages after treatment with SP600125, SB203580, or PDTC detected by RT-qPCR. **(F)** The level of TNF-α and IL-10 in macrophages after treatment with SP600125, SB203580, or PDTC measured by ELISA. *p < 0.05 vs. macrophages treated with DMSO. **(G)** Expression of iNOS and Arg-1 in macrophages after treatment with SP600125, SB203580, or PDTC detected by immunofluorescence staining. *p < 0.05 vs. macrophages treated with DMSO. Measurement data were expressed as mean ± standard deviation. Differences between two groups were compared by unpaired *t*-test. Comparisons of data among multiple groups were conducted by one-way ANOVA. The experiment was repeated three times independently.

Following the results demonstrating that miR-132-3p could regulate MEKK3 to inhibit M1 polarization of macrophages, we further investigated whether miR-132-3p regulated MEKK3 to modulate the downstream NF-κB and p38/JNK signaling pathways so as to affect the macrophage M1 polarization. Western blot analysis suggested that the protein level and phosphorylated protein level of JNK, p38, and IKK-b were downregulated markedly in macrophages transfected with si-MEKK3 and NC inhibitor or transfected with miR-132-3p mimic, compared with macrophages transfected with si-NC and NC inhibitor or transfected with NC mimic, respectively (*p* < 0.05) (Figures 5A,C). Results of dual-luciferase reporter gene assay indicated that the activity of NF-κB was distinctly decreased in macrophages treated with miR-132-3p mimic or both si-MEKK3 and NC inhibitor relative to the macrophages treated with NC mimic or both si-NC and NC mimic independently (*p* < 0.05) (Figures 5B,D). The above findings showed that miR-132-3p could negatively regulate MEKK3 expression to inhibit the activation of the NF-κB and p38/JNK signaling pathways. In order to verify the effects of these signaling pathways on macrophage polarization, macrophages were treated with DMSO, SP600125 (an inhibitor of the JNK signaling pathway), SB203580 (an inhibitor of the p38 signaling pathway), or PDTC (an inhibitor of the NF-κB signaling pathway). The expression of iNOS and TNF-α was found to be decreased and that of IL-10 and Arg-1 was increased in macrophages treated with SP600125, SB203580, or PDTC (*p* < 0.05) (Figure 5E). Moreover, treatment with SP600125, SB203580, or PDTC reduced the level of TNF-α while elevating the IL-10 level in macrophages (*p* < 0.05) (Figure 5F). Immunofluorescence staining suggested a decrease of iNOS level and an enhancement of Arg-1 level in macrophages following treatment with SP600125, SB203580, or PDTC (*p* < 0.05) (Figure 5G). In conclusion, miR-132-3p overexpression can reduce MEKK3 expression and then inactivate the NF-κB and p38/JNK signaling pathways, thus inhibiting M1 polarization of macrophages from SCIR injury rats.

### Overexpression of MiR-132-3p Alleviates SCIR Injury in Rats

**FIGURE 6:**
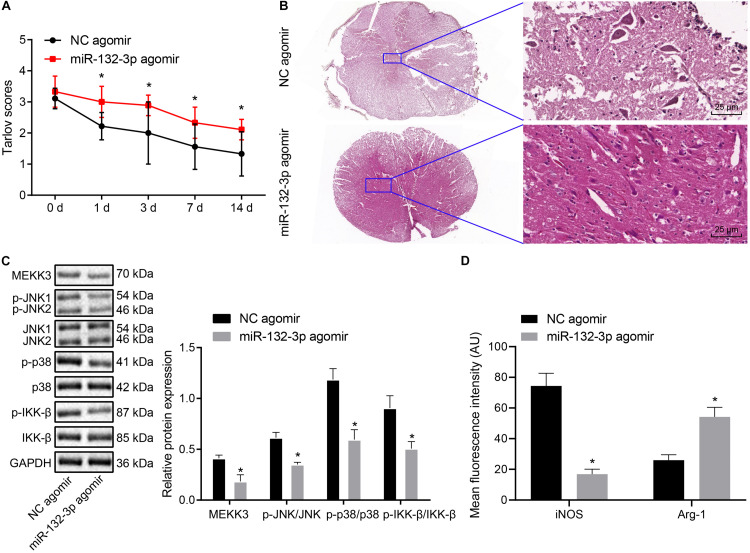
miR-132-3p ameliorates SCIR injury *in vivo*. SCIR injury rats were treated with miR-132-3p agomir or NC agomir. **(A)** Motor function of rats assessed by Tarlov score. **(B)** Histological characteristics of spinal cord tissues of rats observed using HE staining. **(C)** The protein level of MEKK3, JNK, p38, and IKK-β as well as the phosphorylated JNK, p38, and IKK-β levels measured by Western blot analysis in rat spinal cord tissues. **(D)** Expression of iNOS and Arg-1 in spinal cord tissues of rats detected by immunofluorescence staining. *p < 0.05 vs. rats treated with NC agomir. Measurement data were expressed as mean ± standard deviation. *n* = 9 for rats following each treatment. Differences between two groups were compared by unpaired *t*-test. The data in panel **(A)** were analyzed by repeated measures ANOVA.

Finally, we proceeded to characterize the effects of miR-132-3p on the SCIR injury *in vivo*. The Tarlov score of rats treated with miR-132-3p agomir was elevated (*p* < 0.05) (Figure 6A). HE staining used to assess the histological characteristics of spinal cord tissues showed that miR-132-3p agomir alleviated spinal cord injury of rats (Figure 6B). The result of Western blot analysis showed that miR-132-3p agomir treatment led to reduced MEKK3, JNK, p38, and IKK-β protein expression as well as decreased phosphorylated JNK, p38, and IKK-β levels (*p* < 0.05) (Figure 6C). Meanwhile, immunofluorescence staining indicated that iNOS expression was downregulated and conversely, Arg-1 expression was upregulated in spinal cord tissues of rats treated with miR-132-3p agomir (*p* < 0.05) (Figure 6D). The above results indicated that upregulation of miR-132-3p relieved SCIR injury in rats.

## Discussion

Spinal cord ischemia-reperfusion injury is a severe complication of thoracic and abdominal aortic surgery, which can lead to blood-spinal cord barrier dysfunction (Li et al., 2014). The pharmacological adjunct development is delayed because of the complicated mechanism of SCIR injury (Bell et al., 2013). Therefore, there is an urgent clinical need to elucidate the underlying mechanism of SCIR injury. MiRNAs have been widely reported to serve as promising agents to decrease the dysfunction related to SCIR injury and to improve the treatment of SCIR injury (Yan et al., 2012; Nieto-Diaz et al., 2014). Hence, we aimed to investigate the role of miR-132-3p in the biological processes of SCIR injury by regulating MEKK3 and the NF-κB and p38/JNK signaling pathways. The findings in the current study provide evidence that miR-132-3p overexpression downregulates MEKK3 to inactivate the NF-κB and p38/JNK signaling pathways, thereby inhibiting M1 polarization of macrophages and further alleviating SCIR injury.

The results of our study also showed that the expression of miR-132-3p was downregulated in spinal cord tissues of SCIR injury rats. A previous study reported that approximately 200 miRNAs were distinctly downregulated on the 7th day after spinal cord injury in comparison with expression of normal rats (Yunta et al., 2012). Another previous study showed that the expression of miR-124a was downregulated from the 1st to the 7th day after spinal cord injury (Nakanishi et al., 2010). Besides, another study indicated that the expression of miR-494 decreased significantly after spinal cord injury and that increasing miR-494 expression by agomir treatment could facilitate the functional recovery, decrease tissue injury and suppress cell apoptosis in a rat model with spinal cord injury (Zhu et al., 2017). In line with the current results, miR-132 was found to be down-regulated in a LPS-induced rat model of SCI (Zhang et al., 2019). Meanwhile, our present study found that the overexpression of miR-132-3p in SCIR injury could inhibit the M1 polarization of macrophages infiltrating the injury site. Macrophages can be polarized toward a detrimental (M1) condition or a beneficial (M2) condition in the injured central nervous system, and macrophages in injured spinal cord mainly retained the M1 phenotype, which is not conducive to recovery (Kroner et al., 2014). M1 and M2 activation of macrophages has been widely studied in recent years, and activation of M1 macrophages is usually marked by increased pro-inflammatory factors including TNF-α and IL-6 (Essandoh et al., 2016). miR-132 abrogates the enhanced concentrations of IL-6, IL-8, and TNF-α induced by LPS, thus alleviating the resultant neuronal cell inflammatory damage in an *in vitro* model of SCI inflammation injury (Zhang et al., 2019). More importantly, miR-132 has been highlighted to promote M2 polarization instead of M1 polarization in macrophages by targeting various transcription factors and adaptor proteins (Essandoh et al., 2016).

Our study also found that MEKK3 was highly expressed in spinal cord tissues of SCIR injury rats and that it was a target gene of miR-132-3p, which could adversely regulate its expression. Consistent with that finding, miRNAs can interact with the 3′UTR of specific target mRNAs and then result in the inhibition of mRNA degradation or translation (Ivey and Srivastava, 2015). MEKK3 was validated to be a target gene of miR-124m and the expression of MEKK3 was increased in a Parkinson’s disease model induced by 1-methyl-4-phenyl-1,2,3,6-tetrahydropyridine (Yao et al., 2018). MEKK3 expression exhibits a remarkable increase in injured dorsal root ganglia cells, while its downregulation helps to ameliorate the symptoms of SCI (Kong et al., 2019). Li et al. previously demonstrated that MEKK3 induces the secretion of inflammatory factors from macrophages (Kim et al., 2007). Taken together, these data suggest that miR-132-3p inhibits M1 polarization of macrophages to alleviate SCIR injury by downregulating MEKK3.

Furthermore, the present study revealed that miR-132-3p could suppress M1 polarization of macrophages by inactivating the NF-κB and p38/JNK signaling pathways *via* MEKK3 downregulation. A previous study suggested the role of MEKK3 as an activator of the NF-κB reporter gene (Nakamura et al., 2010). MEKK3 downregulation was found to block the transcription activity of NF-κB to further elevate the MCF-7 cell sensitivity to TNF-related apoptosis inducing ligand in breast cancer (Guo et al., 2012). In addition, MEKK3 has the capacity to promote activation of the p38/JNK signaling pathway induced by LPS (Huang et al., 2004). Both MAPK and NF-κB levels are found to correlate with activation and polarization of macrophages, and MAPK phosphorylation led to activation of the NF-κB signaling pathway (Fu et al., 2017). TNF-α, p38, as well as JNK, were revealed to be activated in the spinal cord after acute cardiac injury (Dai et al., 2007). Zhong et al. (2017) reported that activation of the JNK/p38 signaling pathway contributed to M1 polarization of macrophages and inflammation, whereas another study indicated that the JNK pathway plays an important role as a regulator in the macrophage polarization (Zhou et al., 2014). Similarly, another study showed that the activation of JNK and NF-κB signaling pathways was necessary for M1 macrophages polarization induced by glycyrrhizic acid (Mao et al., 2015). Overexpression of miR-132-3p triggers the inhibition of expression of its target FOXO3 and consequently blunts FOXO3-dependent NF-κB signaling pathway activation, thereby attenuating LPS-induced inflammatory response following acute lung injury (Ivey and Srivastava, 2015).

**FIGURE 7:**
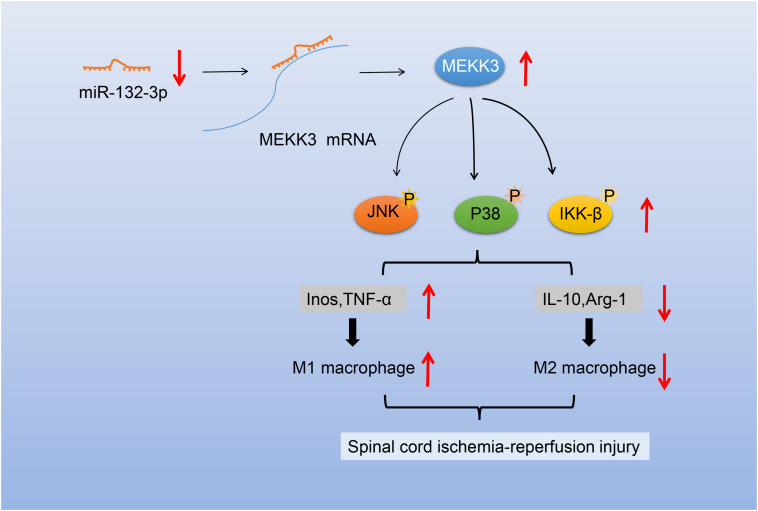
Schematic diagram of the mechanism of miR-132-3p in SCIR injury rats. miR-132-3p targets MEKK3 and negatively regulates the MEKK3 expression to inhibit activation of the NF-κB and p38/JNK signaling pathways, thus inhibiting the M1 polarization of macrophages and further alleviating the motor dysfunction caused by SCIR injury.

Taken together, our study proved that overexpression of miR-132-3p could block the activation of the MEKK3-mediated NF-κB and p38/JNK signaling pathways to inhibit M1 polarization of macrophages, thus alleviating SCIR injury (Figure 7). This fundamental information indicates that the overexpression of miR-132-3p may provide a novel strategy for SCIR injury treatment. In addition to MEKK3, there are numerous other targets for miR-132-3p (Ivey and Srivastava, 2015; Kong et al., 2019), and thus the established correlation between miR-132-3p and MEKK3 in the current study requires further investigation to strengthen the validity of the overall results. We are currently investigating the specific site regulating the correlation of miR-132-3p, MEKK3, and NF-κB and p38/JNK signaling pathways in SCIR injury.

## Data Availability Statement

The original contributions presented in the study are included in the article/Supplementary Material, further inquiries can be directed to the corresponding author/s.

## Ethics Statement

The animal study was reviewed and approved by the Experimental Animal Ethics Committee of Guizhou Provincial People’s Hospital.

## Author Contributions

HF and H-FL designed the study. QP, H-LJ, and MY carried out the experiments. R-RW, Q-YW, and J-PZ analyzed the data. HF, H-FL, QP, and H-LJ made the Figures. All authors were involved in the drafting, reading, and revision of the manuscript and approved the final manuscript. All authors read and approved the final manuscript.

## Conflict of Interest

The authors declare that the research was conducted in the absence of any commercial or financial relationships that could be construed as a potential conflict of interest.

## Footnotes

1 http://www.targetscan.org/vert_71/

## Abbreviations

SCIR: spinal cord ischemia-reperfusion
MiR-132-3p: microRNA-132-3p
MiRNAs or miRs: microRNAs
MEKK3: mitogen-activated protein kinase kinase kinase 3
NF-κB: nuclear factor-kappaB
TNF-α: tumor necrosis factor- α
iNOS: inducible nitric oxide synthase
IL-6: interleukin-6
JNK: c-JunN-terminal kinase
RPMI: Roswell Park Memorial Institute
LPS: lipopolysaccharide
NC: negative control
HE: hematoxylin-eosin
PBS: phosphate buffered saline
BSA: bovine serum albumin
PDTC: pyrrolidine dithiocarbamate
RT-qPCR: reverse transcription quantitative polymerase chain reaction
GADPH: glyceraldehyde-3-phosphate dehydrogenase
cDNA: complementary DNA
RIPA: radio-immunoprecipitation assay
HRP: horseradish peroxidase
IgG: immunoglobulin G
ELISA: enzyme-linked immunosorbent assay
IL-10: interleukin-10
OD: optical density
WT: wild type
MUT: mutant type
3′UTR: 3 ′ untranslated region
HEK-293T: human embryonic kidney 293T
IKK-β: I κ B kinase complex
Arg-1: arginase-1
ANOVA: analysis of variance
PP2A: protein phosphatase 2A.
