# Supplementary material for: MiR-132-3p Modulates MEKK3-Dependent NF-κB and p38/JNK Signaling Pathways to Alleviate Spinal Cord Ischemia-Reperfusion Injury by Hindering M1 Polarization of Macrophages
Source: Front Cell Dev Biol. 2021 Feb 11;9:570451. doi: 10.3389/fcell.2021.570451 (PMC7905026; doi:10.3389/fcell.2021.570451)
Supplement: Supplementary file 1 [file Table_1.DOCX]

fig3B:



GAPDH



MEKK3

fig3G:



GAPDH



MEKK3

fig6C:



GAPDH



IKK-β



JNK



MEKK3



p38



p-IKK-β



p-JNK



p-p38
